# A Monocular SLAM-based Controller for Multirotors with Sensor Faults under Ground Effect

**DOI:** 10.3390/s19224948

**Published:** 2019-11-13

**Authors:** Antonio Matus-Vargas, Gustavo Rodriguez-Gomez, Jose Martinez-Carranza

**Affiliations:** 1 Department of Computational Science, Instituto Nacional de Astrofísica, Óptica y Electrónica (INAOE), Puebla 72840, Mexico; matusv@inaoep.mx (A.M.-V.); grodrig@inaoep.mx (G.R.-G.); 2 Department of Computer Science, University of Bristol, Bristol BS8 1UB, UK

**Keywords:** multirotor, ground effect, sensor faults

## Abstract

Multirotor micro air vehicles can operate in complex and confined environments that are otherwise inaccessible to larger drones. Operation in such environments results in airflow interactions between the propellers and proximate surfaces. The most common of these interactions is the ground effect. In addition to the increment in thrust efficiency, this effect disturbs the onboard sensors of the drone. In this paper, we present a fault-tolerant scheme for a multirotor with altitude sensor faults caused by the ground effect. We assume a hierarchical control structure for trajectory tracking. The structure consists of an external Proportional-Derivative controller and an internal Proportional-Integral controller. We consider that the sensor faults occur on the inner loop and counteract them in the outer loop. In a novel approach, we use a metric monocular Simultaneous Localization and Mapping algorithm for detecting internal faults. We design the fault diagnosis scheme as a logical process which depends on the weighted residual. Furthermore, we propose two control strategies for fault mitigation. The first combines the external PD controller and a function of the residual. The second treats the sensor fault as an actuator fault and compensates with a sliding mode action. In either case, we utilize onboard sensors only. Finally, we evaluate the effectiveness of the strategies in simulations and experiments.

## 1. Introduction

The interest in Unmanned Aerial Vehicles (UAVs) has been growing in recent years. Different types of UAVs have been used in many applications such as photography, cinematography, surveillance, remote inspection and emergency response, to mention a few. Among the types of UAVs, rotary-wing vehicles are one of the most versatile platforms. Particularly, multirotors offer large payload, high mobility and simple construction.

Micro Air Vehicles (MAVs) equipped with onboard sensors are ideal platforms for autonomous navigation. Due to payload and energy restrictions, an MAV is equipped with fewer sensors. The minimal sensor suite for autonomous localization has been reported to be a monocular camera and an Inertial Measurement Unit (IMU) [1]. However, it is common that an MAV is also equipped with sensors to measure the altitude. For low-level flights, the altitude is obtained from a combination of a range sensor and a barometer; this combination is referred to as an altimeter. With a monocular camera and the altitude measurement, the autonomous flight of a quadrotor has been achieved for indoor scenarios [2].

Multirotor MAVs can operate in complex and confined environments that are not accessible to larger drones. Within such environments, a multirotor is likely to move close to horizontal and vertical surfaces. These situations result in airflow interactions between the propellers and proximate surfaces. The most common of these interactions is the one produced by the ground. This phenomenon, known as the ground effect, is more pronounced in rotorcraft operating in hover and at low speeds. In addition to the increment of thrust efficiency, it has been reported that the ground effect causes variations in the pressure altitude reported by the barometer [3].

The ground effect has been well researched for helicopters [4,5,6]. For multirotors, empirical models have been developed [7,8]. Furthermore, several control schemes have been proposed for compensating the ground effect in quadrotors such as adaptive [9], fuzzy [10], sliding mode [7] and multi-controller [11]. However, the studies above have only considered the case in which there are no faults in the multirotor. Those controllers are not suitable for operating the vehicle in the case of actuator or sensor faults.

When there is no fault, a hierarchical control structure, composed by an external Proportional-Derivative (PD) controller and an internal Proportional-Integral (PI) controller, is developed for trajectory tracking. In contrast to previous work, we do not use external sensors to observe system states such as the vehicle’s pose. Instead, we employ a technique known as *Monocular Simultaneous Localization and Mapping* (Monocular SLAM) to observe the position and orientation of the multirotor. The measurement of these states are estimated with metric, thus enabling us to implement position feedback for the external PD controller; the internal PI controller receives measurements from the altimeter. Sensor faults occur on the inner loop and we counteract them in the outer loop. We design the fault detection unit as a logical process that depends on the weighted residual. The residual compares estimations from the SLAM system with faulty internal readings. When a sensor fault is identified, the system switches to a control sub-law. We derive two control sub-laws—the first combines the external PD controller and a function of the residual; the second treats the sensor fault as an actuator fault and compensates with a sliding mode action. To the authors’ knowledge, this is the first time that sensor faults caused by the ground effect are tackled utilizing a state estimator based on monocular SLAM. The latter is a well-known technique in robotics, typically used to estimate a robot’s pose, but it has never been used to address any issues related to ground effect in multirotors.

The organization of the rest of the paper is as follows. Section 2 presents the related literature considering the ground effect in multirotors. In Section 3, we propose the fault detection unit and the control sub-laws. Section 4 discusses the simulation and experimental results. Finally, conclusions are given in Section 5.

## 2. Related Work

For safety reasons, full-scale helicopters are not allowed to operate close to obstacles. However, operating close to the ground is unavoidable. This circumstance has motivated the study of aerodynamic interactions between the helicopter and the ground while ignoring other surfaces like walls and the ceiling. As a result, the most known of these interactions is the ground effect. Simplified models for one rotor in ground effect were proposed, such as the model of Cheeseman [4] and the model of Hayden [5]. The applicability of these models to micro-rotors has been questioned because they operate at significantly different Reynolds numbers [12]. On the other hand, the wake flow of a single micro-rotor in ground effect has been studied thoroughly [6,13,14]. For a small helicopter, an empirical formula for ground compensation has been proposed [7]. Though simple, the generalization of this formula to other vehicles is not clear.

The effect of the ground on multirotor MAVs has been acknowledged in several works. An earlier approach was to model this effect as a variable thrust coefficient, which could be adapted on-line [9,10,15]. Alternatively, a method for modeling the same effect on a quadrotor used visual feedback from streamers attached to the ground [8]. Here, the aggregate energy due to the quadrotor down-wash was calculated and combined with the current throttle to predict the ground effect. The limitation of this work is that it requires streamers on the ground. In Reference [16], the model of Cheeseman has been compared to empirical data collected with a micro quadrotor, showing that the ground effect manifested at a higher height than predicted by the model. Similarly, a series of experiments was conducted to juxtapose the same model and data obtained from a coaxial quadrotor [17]. Again, the results pointed to a stronger ground effect than predicted by the theory. Motivated by this, the model of Cheeseman was extended for a quadrotor in Reference [18]. The authors also introduced the partial ground effect, which appears when only some of the rotors experience the ground effect. Naturally, the aforementioned models have been combined with control techniques [19,20,21].

When a multirotor flies over an obstacle, it is influenced by the ground effect. Considering that a depth image of the obstacle is available, the impulse affecting a quadrotor that is passing over that obstacle has been predicted from prior experience [22]. It was demonstrated that the aerodynamic interactions between the vehicle and the local environment produce consistent effects. The prediction scheme was nevertheless prone to either overestimating or underestimating these effects. This scheme was later incorporated into the control loop [23]. With this approach, a significant improvement was achieved for seen obstacles. For unobserved objects, the disturbance was not properly compensated. A similar problem was attacked in Reference [18], where the form of the obstacle was restricted to a box.

A state observer incorporates a mathematical model of the quadrotor and can be used to estimate external disturbances. The estimation can be combined with a feedback controller. This combination has been reported to improve the control performance when external disturbances exist [24,25,26,27]. However, the observer’s estimation deteriorates for small disturbances and large sensor noise. Filtering the inputs and outputs of the observer can help in this case. The tuning of the filters is intricate and the filters introduce delays in the estimation, which deteriorates the overall control performance. On the contrary, stochastic estimation algorithms are designed to handle process and sensor noise at the cost of increasing the computational burden. An unscented Kalman filter has been presented to estimate external force and torque for quadrotors [28]. Nevertheless, it is not clear if this approach can be applied to disturbance rejection.

In Reference [11], we have shown that the position control performance under the ground effect can be enhanced with a rapid switching control algorithm. The position feedback was provided by an external motion capture system at a high frequency. We proposed a multi-controller structure where a classical controller and an extra one with a firm variable action were combined. The latter was designed to take action when the vehicle was not responding to the former. We treated these situations as mere disturbances caused by the increment of thrust in the ground effect. However, we noticed that sometimes the vehicle was suddenly starting to descend without being commanded to do so. This observation motivated us to check for possible faults in the drone. Indeed, we found spurious altimeter faults in the ground effect (see Section 4.1). Even though our previous approach did improve the control performance, there was still room for improvement, especially in avoiding reliance on external sensors.

The literature mentioned above has only considered the case in which faults do not occur in the multirotor. In the case of actuator or sensor faults, conventional controllers are not suitable for operating the vehicle due to significant control errors. In Reference [29], the research on fault diagnosis and fault-tolerant control for quadrotors was reviewed. Little research has examined the fault diagnosis and control for multirotors with sensor faults. In particular, the literature comprises faults in sensors like accelerometers, magnetometers and rate gyros [30,31,32]. More recently, the work in Reference [33] has considered faults in the velocity measurement obtained from the Global Positioning System (GPS) and the Inertial Navigation System (INS). One observer was designed for detection and another to estimate the sensor fault. Under several constraints, the estimation error was proved to be uniformly ultimately bounded. The bound is directly proportional to the magnitude of the derivative of the sensor fault. This fact might limit the effectiveness of such an observer since the sensor faults under the ground effect tend to have rapid changes. Furthermore, this approach considered faults occurring in the same loop.

In summary, the literature is lacking in terms of the diagnosis and control of sensor faults for multirotors. Furthermore, this is the first time that sensor faults induced by the ground effect have been reported. Consequently, the first attempts at mitigating these faults are going to be presented in this paper.

## 3. Methods and Materials

This section starts with a summary of the multirotor dynamics model. Subsequently, a specific control scheme is assumed and the stability conditions for the fault-free system are shown analytically. Finally, the fault diagnosis scheme and two control sub-laws are proposed.

### 3.1. Multirotor Dynamics

**Figure 1:**
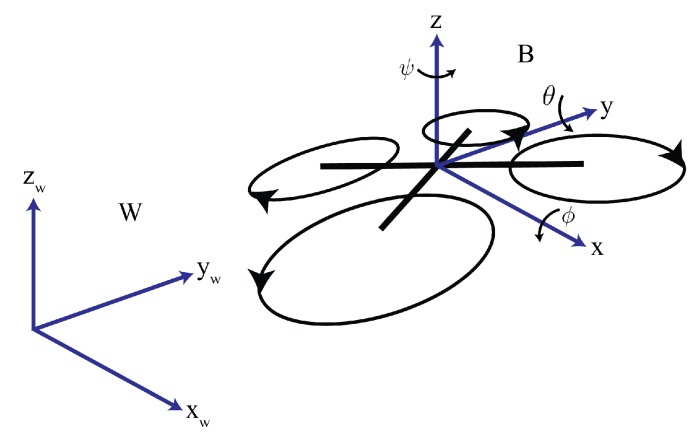
Coordinate frames definition.

The mathematical model of the multirotor is deduced by introducing a world-fixed coordinate system {W} and a body-fixed coordinate system {B} (see Figure 1). By using either the Newton-Euler equations or the Euler-Lagrange formalism, the model of the multirotor can be obtained [34,35,36]. We selected the following model to describe the multirotor unmanned helicopter:

(1) $$\begin{array}{cc} \ddot{x}\left(t\right) & =u_{z}\left(t\right)\left(\sin\psi \sin\phi +\cos\psi \sin\theta \cos\phi \right)/m\\ \ddot{y}\left(t\right) & =u_{z}\left(t\right)\left(\sin\psi \sin\theta \cos\phi -\cos\psi \sin\phi \right)/m\\ \ddot{z}\left(t\right) & =u_{z}\left(t\right)\cos\theta \cos\phi /m-g\\ \ddot{\phi }\left(t\right) & =u_{\phi }\left(t\right)/J_{x}\\ \ddot{\theta }\left(t\right) & =u_{\theta }\left(t\right)/J_{y}\\ \ddot{\psi }\left(t\right) & =u_{\psi }\left(t\right)/J_{z} \end{array},$$

where ${\left[x,y,z\right]}^{T}\in R^{3}$ is the position vector from the origin of the body frame to the origin of the world frame, ${\left[\phi ,\theta ,\psi \right]}^{T}\in R^{3}$ is the vector of Euler angles (roll, pitch, and yaw), m∈R is the mass of the vehicle, $\mathrm{diag}\left(J_{x},J_{y},J_{z}\right)\in R^{3\times 3}$ is the inertia matrix, g∈R is the acceleration of gravity and ${\left[u_{z},u_{\phi },u_{\theta },u_{\psi }\right]}^{T}\in R^{4}$ is the vector of control inputs.

Assuming that the rotorcraft moves around the hovering state, the model (1) reduces to:

(2) $$\begin{array}{cc} \ddot{x}\left(t\right) & =\theta (t)g\\ \ddot{y}\left(t\right) & =-\phi (t)g\\ \ddot{z}\left(t\right) & =u_{z}\left(t\right)/m-g\\ \ddot{\phi }\left(t\right) & =u_{\phi }\left(t\right)/J_{x}\\ \ddot{\theta }\left(t\right) & =u_{\theta }\left(t\right)/J_{y}\\ \ddot{\psi }\left(t\right) & =u_{\psi }\left(t\right)/J_{z} \end{array}.$$

From (2), it is clear that the translational axes are decoupled. Thus, the *z*-axis dynamic model of the multirotor can be separated as follows:

(3) $$\ddot{z}\left(t\right)=u_{v}\left(t\right).$$

This model implies that any possible gravity is subsumed under the $u_{v}$ term:

(4) $$\ddot{z}\left(t\right)=\frac{u_{z}\left(t\right)}{m}-g=u_{v}\left(t\right),$$

where $u_{z}\left(t\right)=m\left[\ddot{z}\left(t\right)+g\right]$ is the actual upward thrust generated by the rotorcraft in Newtons. In control system theory, it is common to compensate factors such as gravity and the mass into $u_{v}$, as the calculation of $u_{z}$ is straightforward and does not depend on any state variables.

### 3.2. Altitude Control

Before developing the fault-free control scheme, the following assumption is required. Define $z_{ref}\left(t\right)\in R$ as the trajectory of reference. This trajectory satisfies that ${\dot{z}}_{ref}\left(t\right)\in R$ and ${\ddot{z}}_{ref}\left(t\right)\in R$ exist for all t≥0. This assumption is reasonable since, in practice, the trajectory that a multirotor can track is limited by the physical attributes of the multirotor.

We follow a cascade control scheme, where a low-level controller is present as the internal loop and a trajectory tracking controller is running as the external loop [37]. The inner loop controls the velocity at a high frequency and the outer loop controls the position at a low frequency. When there are no sensor faults, the formulation of the latter is:

(5) $$u_{p}\left(t\right)=k_{p,1}\left[z_{ref}\left(t\right)-z\left(t\right)\right]+k_{d,1}\frac{d}{dt}\left[z_{ref}\left(t\right)-z\left(t\right)\right]+{\dot{z}}_{ref}\left(t\right),$$

where $k_{p,1}\in R$ and $k_{d,1}\in R$ are controller parameters to be designed. The reference velocity for the inner loop controller is obtained simply as $v_{ref}=u_{p}$. Then, the formulation of this loop’s controller is: title = Active Fault-Tolerant Control for a Quadrotor with Sensor Faults,

(6) $$u_{v}\left(t\right)=k_{p,2}\left[v_{ref}\left(t\right)-v_{z}\left(t\right)\right]+k_{i,2}\int_{0}^{t}\left[v_{ref}\left(t\right)-v_{z}\left(t\right)\right]d\tau +k_{d,2}\frac{d}{dt}\left[v_{ref}\left(t\right)-v_{z}\left(t\right)\right]$$

where $k_{p,2}\in R$, $k_{i,2}\in R$, and $k_{d,2}\in R$ are controller parameters and $v_{z}\left(t\right)\in R$ is the *z*-axis velocity of the robot.

Define the position and velocity errors as $e_{z}=z_{ref}-z$ and $e_{v}=v_{ref}-v_{z}$, respectively. The following two propositions provide the stability conditions for the outer and inner controllers.

**Proposition** **1.** *The position error $e_{z}$, resulting from the application of the external controller (5) to the model (3), converges asymptotically to zero if $k_{p,1}/\left(1+k_{d,1}\right)>0$.*

**Proof.**  Given that the internal loop is faster than the external one, we can regard the model from $u_{p}$ to $\dot{z}$ as a proportion. Therefore, recalling the form of $e_{z}$, we have that:

(7) $${\dot{e}}_{z}\left(t\right)={\dot{z}}_{ref}\left(t\right)-u_{p}=-k_{p,1}e_{z}-k_{d,1}{\dot{e}}_{z}=-\frac{k_{p,1}}{1+k_{d,1}}e_{z}.$$

The characteristic equation of (7) is:

(8) $$\lambda +\frac{k_{p,1}}{1+k_{d,1}}=0.$$

Since $k_{p,1}/\left(1+k_{d,1}\right)>0$., the only root of (8) is negative, which implies that (7) is stable and $e_{z}\left(t\right)$ reaches zero asymptotically. □

**Proposition** **2.** *The velocity error $e_{v}$, resulting from the application of the internal controller (6) to the model (3), converges asymptotically to zero if $k_{p,2}/\left(1+k_{d,2}\right)>0$ and $k_{i,2}/\left(1+k_{d,2}\right)>0$.*

**Proof.**  For small time steps, it is reasonable to assume constant acceleration between time steps, which means that ${\ddot{v}}_{ref}\left(t\right)=0$. Then, recalling the form of $e_{v}$, we have that:

(9) $$\begin{array}{cc} {\dot{e}}_{v}\left(t\right) & ={\dot{v}}_{ref}\left(t\right)-{\dot{v}}_{z}\left(t\right)\\ {\ddot{e}}_{v}\left(t\right) & ={\ddot{v}}_{ref}\left(t\right)-{\dot{u}}_{v}\left(t\right)=-k_{p,2}{\dot{e}}_{v}\left(t\right)-k_{i,2}e_{v}\left(t\right)-k_{d,2}{\ddot{e}}_{v}\left(t\right) \end{array}.$$

Then, the system can be rewritten as:

(10) $$\left[\begin{array}{c} {\dot{e}}_{v}\left(t\right)\\ {\ddot{e}}_{v}\left(t\right) \end{array}\right]=\left[\begin{array}{cc} 0 & 1\\ -\frac{k_{i,2}}{1+k_{d,2}} & -\frac{k_{p,2}}{1+k_{d,2}} \end{array}\right]\left[\begin{array}{c} e_{v}\left(t\right)\\ {\dot{e}}_{v}\left(t\right) \end{array}\right].$$

The characteristic equation of (10) is:

(11) $$\lambda^{2}+\frac{k_{p,2}}{1+k_{d,2}}\lambda +\frac{k_{i,2}}{1+k_{d,2}}=0.$$

Since $k_{p,2}/\left(1+k_{d,2}\right)>0$ and $k_{i,2}/\left(1+k_{d,2}\right)>0$, the real parts of the roots of (11) are negative, which implies that the system (10) is stable and $e_{v}\left(t\right)$ reaches zero asymptotically. □

In addition to the conditions in Propositions 1 and 2, the parameters of the external and internal controllers should be determined according to the real circumstances of the multirotor under consideration. Particularly, it is common to use only PI control in the inner loop of multirotors [38]. This choice is justified in the stability analysis, where the derivative gain appears, dividing the others, meaning that it slows down the internal closed-loop dynamics.

### 3.3. Metric Monocular SLAM

External sensors for localization of the drone are not adequate for complex environments. To overcome this restriction, we can use a visual SLAM method. Given a monocular onboard camera, ORB-SLAM2 can be employed to obtain the camera pose and 3D point estimates without metric [39]. To address the scale problem, assuming planar ground and knowing the camera angle and distance to the ground, we can obtain a synthetic depth image by resolving the ray-ground intersection geometry. This synthetic image can be coupled with incoming RGB images and then fed to the RGB-D version of ORB-SLAM2, which generates metric pose estimates [2].

**Figure 2:**
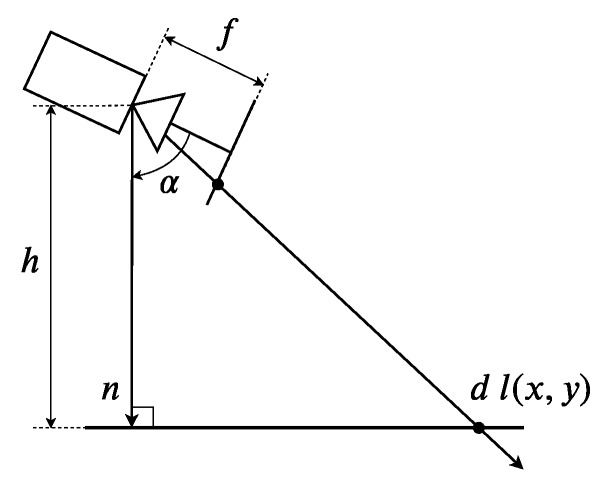
Geometric configuration to generate the synthetic depth image.

**Figure 3:**
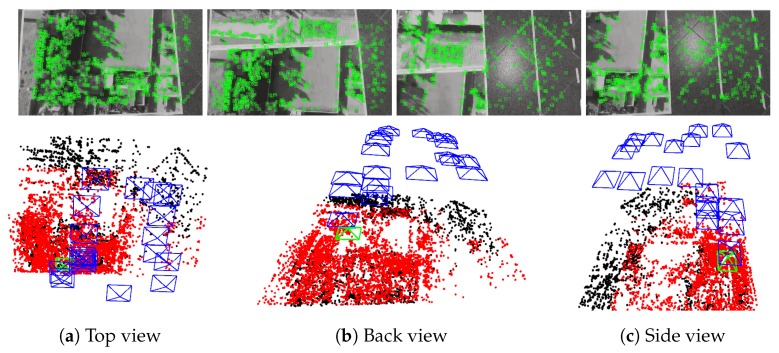
Snapshots that illustrate the functioning of the metric monocular SLAM; the top row provides samples from camera frames with the tracked features (green markers); the bottom row displays three views of the map with keyframes (blue pyramids, the first one is green) and map points (red ones are being used for tracking, while black ones are ignored).

Figure 2 illustrates the side view of the ray-ground geometric configuration. Knowing the height above ground of the camera *h* and the camera angle α, we can define a vector *n* perpendicular to the ground. Therefore, a vector *l* departing from the camera’s optical center $\left(x_{0},y_{0}\right)$ and passing through a pixel (x,y) will intersect the planar ground for some scalar *d*. The value of *d* is obtained with the ray-plane intersection equations:

(12) $$\begin{array}{cc} l= & {\left[\left(x_{0}-x\right)/f,\left(y_{0}-y\right)/f,1\right]}^{T}\\ n= & {\left[0,-h\sin\alpha ,h\cos\alpha \right]}^{T}\\ d= & \frac{n^{T}\cdot n}{l^{T}\cdot n} \end{array},$$

where *f* is the focal length. This approach has been evaluated using the Vicon motion capture system in indoor environments. It was found that the pose error is 2% on average [2]. Figure 3 shows examples of the system carrying out the metric mapping. In this figure, it is noticed that all map points (red and black, obtained from the synthetic depth map) are on a three dimensional plane, which corresponds to the ground.

The height above ground of the camera can be obtained from the altimeter of the drone. The camera angle can be set a priori either via hardware or via software if its field of view can be foveated. We propose using the metric monocular SLAM system because it offers the following advantages. First, the metric monocular SLAM system is suitable for autonomous navigation, which means that such a system is likely to be already present in the control architecture. Second, if correctly initialized, it is robust against erroneous measurements of altitude (see Section 4.1). This property holds since the original SLAM algorithm at startup creates a keyframe with the first frame, sets its pose as the origin and creates an initial map from all keypoints with depth information [39]. The system optimizes the camera pose by finding features matches to the local map. It also optimizes the entire map by keeping the origin keyframe fixed. Therefore, the initial synthetic depth image should be generated with faultless altitude readings, which are easily obtained while hovering far from horizontal and vertical surfaces.

### 3.4. Fault-tolerant Control

**Figure 4:**
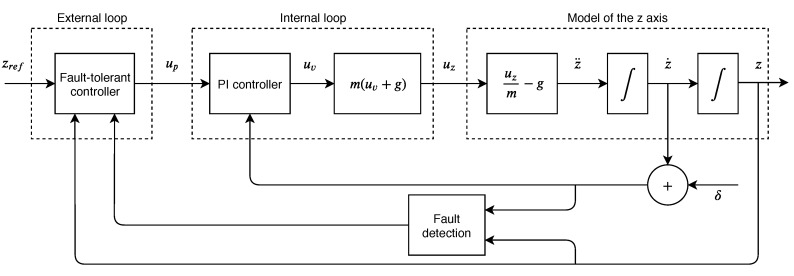
Fault-tolerant control schematic.

We utilize a fault-tolerant control architecture. Figure 4 illustrates this architecture applied to our problem. First, we will describe the interior of the fault detection unit. We assume that there are available three measurements related to the altitude of the drone—range-based ($y_{r}$), inertial-based ($y_{i}$), and vision-based ($y_{v}$). The first measurement can be obtained from a combination of a range sensor (ultrasonic, infrared, laser) and a barometer. The second is commonly obtained from the IMU. The last is from the vision algorithms which use an onboard camera. These measurements relate to the state as:

(13) $$\begin{array}{cc} y_{r}\left(t\right) & =z(t)+\delta (t)\\ y_{i}\left(t\right) & =\dot{z}\left(t\right)\\ y_{v}\left(t\right) & =z(t) \end{array},$$

in which δ(t)∈R is the sensor fault. Let us consider $r\left(t\right)\in R^{2}$ as the residual vector defined as follows:

(14) $$r\left(t\right)=\left[\begin{array}{c} r_{1}\left(t\right)\\ r_{2}\left(t\right) \end{array}\right]=\left[\begin{array}{c} y_{r}\left(t\right)-y_{v}\left(t\right)\\ {\dot{y}}_{r}\left(t\right)-y_{i}\left(t\right) \end{array}\right].$$

Next, we define the residual evaluation function $J_{eval}\left(t\right)\in R$ with the formulation below:

(15) $$J_{eval}\left(t\right)={\left[r^{T}\left(t\right)Wr\left(t\right)\right]}^{1/2}={\left||r\left(t\right)|\right|}_{W},$$

where $W\in R^{2\times 2}$ is a real symmetric positive definite weighting matrix. In the literature, the fault diagnosis relies only on the norm of the residual [40]. We propose the weighting matrix to select the relative importance of the different components of the residual vector. Thus, the fault detection unit is operated by a logical process:

(16) $$d_{lp}=\left\{\begin{array}{cc} J_{eval}\left(t\right)\leq J_{th} & \mathrm{normal}\;\mathrm{case}\\ J_{eval}\left(t\right)>J_{th} & \mathrm{fault}\;\mathrm{case} \end{array}\right.,$$

where $J_{th}\in R$ is the threshold of the fault detection unit and its value can be designed according to the experience of experts, considering uncertainties and disturbances present in the system.

So far, the detection or diagnosis scheme has been presented. In what follows, we will describe the general form of the fault-tolerant control law. Then, we will derive two control sub-laws that will be activated when a sensor fault is identified; the first deals with the sensor fault directly and the second deals with it as an actuator fault.

In general, the objective is to find a fault-tolerant control law for the external loop like the following equation:

(17) $$u_{p}\left(t\right)=\left\{\begin{array}{cc} u_{p}^{n}\left(t\right) & \mathrm{normal}\;\mathrm{case}\\ u_{p}^{f}\left(t\right) & \mathrm{fault}\;\mathrm{case} \end{array}\right..$$

Inserting the measurement, $u_{p}^{n}$ will always remain as:

(18) $$u_{p}^{n}\left(t\right)=k_{p,1}\left[z_{ref}\left(t\right)-y_{v}\left(t\right)\right]+k_{d,1}\frac{d}{dt}\left[z_{ref}\left(t\right)-y_{v}\left(t\right)\right]+{\dot{z}}_{ref}\left(t\right).$$

The idea is to design $u_{p}^{f}$ such that the multirotor stays close to the trajectory of the reference whenever a sensor fault is identified. If the magnitude of the sensor fault is small, the fault will not be detected by our fault detection unit. In this case, the control law (18) still guarantee the stability of the multirotor though the tracking error will not stay close to zero.

For the first control sub-law, suppose the inner loop uses a measurement with the form of the range sensor measurement in (13):

(19) $$u_{v}\left(t\right)=k_{p,2}\left[v_{ref}\left(t\right)-{\dot{y}}_{r}\left(t\right)\right]+k_{i,2}\int_{0}^{t}\left[v_{ref}\left(t\right)-{\dot{y}}_{r}\right]d\tau .$$

For compensating disturbances produced by sensor faults of the inner loop, we will design an additive term $u_{a}\in R$ for the outer loop. Taking into account the sensor fault and the additive term, $u_{v}$ becomes:

(20) $$u_{v}\left(t\right)=k_{p,2}e_{v}\left(t\right)+k_{i,2}\int_{0}^{t}e_{v}\left(t\right)d\tau +k_{p,2}\left[u_{a}-\dot{\delta }\left(t\right)\right]+k_{i,2}\int_{0}^{t}\left[u_{a}-\dot{\delta }\left(t\right)\right]d\tau .$$

From the previous equation, it is clear that $u_{a}$ should be designed to be equal to $\dot{\delta }$. However, the time derivative of δ can be computed either with respect to the external loop or to the internal loop. To justify the selection, we will analyze the perturbation term $p=u_{a}-\dot{\delta }$. Let $T_{p}$ and $T_{v}$ be the sampling periods of the external and internal loops, respectively. We assume that $\mu =T_{p}/T_{v}\in Z^{+}$. In discrete form, after one step of $T_{p}$, it yields:

(21) $$p=\sum_{j=k-\mu +1}^{k}\left(u_{a}-\frac{\delta_{j}-\delta_{j-1}}{T_{v}}\right),$$

where the subscript of δ denote the discrete-time index, in essence, $\delta_{k}=\delta \left(kT_{v}\right)$. The expansion of the summation results in:

(22) $$\begin{array}{ll} p= & \left(u_{a}-\frac{\delta_{k}-\delta_{k-1}}{T_{v}}\right)+\left(u_{a}-\frac{\delta_{k-1}-\delta_{k-2}}{T_{v}}\right)+\cdots \\ & +\left(u_{a}-\frac{\delta_{k-\mu +2}-\delta_{k-\mu +1}}{T_{v}}\right)+\left(u_{a}-\frac{\delta_{k-\mu +1}-\delta_{k-\mu }}{T_{v}}\right)=\frac{T_{p}u_{a}-\left(\delta_{k}-\delta_{k-\mu }\right)}{T_{v}}. \end{array}$$

The last form of *p* implies that the additive term should be $u_{a}=\left(\delta_{k}-\delta_{k-\mu }\right)/T_{p}$. This means that internal sensor faults can be countered with information available at the time-steps of the external loop, no matter the values in between (at the cost of an approximation error). This is useful if the external loop receives little information about the internal one, which is likely the case when working with off-the-shelf drones. In reality, δ cannot be measured directly. For this reason, we propose the following expression:

(23) $$u_{a}\left(t\right)=k_{a}r_{2}\left(t\right).$$

If both loops were to run at the same frequency, we would have nearly total fault cancellation with $k_{a}=1$ since $r_{2}\approx \dot{\delta }$. However, it is important to remember that the inner loop executes faster than the outer loop, so $u_{a}$ remains constant until a new outer loop command is computed. In this case, choosing unitary $k_{a}$ will result in an overcompensation of the fault. Moreover, the value of $r_{2}$ could be contaminated with noise. Therefore, we propose the range of this parameter to be $0<k_{a}<1$. Furthermore, this parameter could take different values for positive and negative disturbances, $k_{a}=\left\{k_{a}^{+},k_{a}^{-}\right\}$. In the end, the first control sub-law is given by following equation:

(24) $$u_{p}^{f}\left(t\right)=k_{p,1}\left[z_{ref}\left(t\right)-y_{v}\left(t\right)\right]+k_{d,1}\frac{d}{dt}\left[z_{ref}-y_{v}\left(t\right)\right]+{\dot{z}}_{ref}\left(t\right)+u_{a}\left(t\right).$$

Some literature has examined converting sensor faults into actuator faults through a transformation [41,42]. For comparison purposes, we consider sensor faults as actuator faults or disturbances and propose a second control sub-law based on Sliding Mode Control (SMC). Before going into detail, some remarks are in order. The time response of the internal loop is much smaller than that of the external loop, which means that the control of velocity is faster than that of the position. Therefore, we can neglect the response time of the velocity control and consider the model from $u_{v}$ to $\ddot{z}$ as a proportion. Thence, the model from $u_{p}$ to $\ddot{z}$ can be regarded as a double integrator. Also, we propose to activate the SMC only when a fault is detected, so $u_{p}\to u_{p}^{f}$. Considering disturbances or the actuator fault, the model of the *z* axis results in:

(25) $$\ddot{z}\left(t\right)=u_{p}^{f}\left(t\right)+c\left(t\right),$$

where c(t) is a composite disturbance term. We assume that |c(t)|≤C for some known C>0. Define the sliding surface *s* with:

(26) $$s\left(t\right)={\dot{e}}_{z}\left(t\right)+k_{e}e_{z}\left(t\right),\;k_{e}>0.$$

The second control sub-law becomes:

(27) $$u_{p}^{f}\left(t\right)={\ddot{z}}_{ref}\left(t\right)+k_{e}{\dot{e}}_{z}\left(t\right)+k_{s}\mathrm{sign}\left[s\left(t\right)\right],\;k_{s}>0.$$

**Proposition** **3.** *The control (27) drives the position error to the sliding surface (26) and keeps the error on the surface thereafter in the presence of the bounded disturbance c(t).*

**Proof.**  Recalling the position error $e_{z}$, it follows that:

(28) $${\ddot{e}}_{z}={\ddot{z}}_{ref}-\ddot{z}={\ddot{z}}_{ref}-u_{p}^{f}-c.$$

Let us select a candidate Lyapunov function as:

(29) $$V=\frac{1}{2}s^{2}.$$

Now, let us take the derivative of *V* as in the usual Lyapunov method:

(30) $$\dot{V}=s\dot{s}=s\left({\ddot{z}}_{ref}-u_{p}^{f}-c+k_{e}{\dot{e}}_{z}\right)=-s\left[k_{s}\mathrm{sign}\left(s\right)+c\right]=-k_{s}\left|s\right|-sc.$$

Taking the absolute value of the second term and considering the disturbance bound, it results in:

(31) $$\dot{V}\leq -k_{s}\left|s\right|+|s|C\leq -\left|s\right|\left(k_{s}-C\right).$$

Suppose $k_{s}$ is chosen appropriately ($k_{s}>C$), it yields that $\dot{V}<0$ for s≠0. Therefore, the region s=0 must be invariant. □

Throughout the next section, we will refer as (Fault-Tolerant Controller) FTC 1 to the strategy that adopts (24), and as FTC 2 to the one that uses (27).

### 3.5. Equipment

For safety, we first tested the proposed control laws in simulation. The whole system was implemented in Simulink. The block diagram approach makes it easy to combine continuous models with discrete systems, such as control loops running on computers. In this way, we can select the sampling period of the external and internal loops individually. Sensor measurements are obtained by simply passing the signals of the plant through zero-order-hold blocks. Also, we emulate the range sensor faults by adding the output of the uniform random number block to the position variable at specified times and intervals.

In the real-world experiments, we used a Parrot Bebop 2 quadcopter for which a software driver is available, allowing the usage of simple commands for takeoff, landing and piloting. The Parrot Bebop 2 weighs 0.5 kg and has a 29 cm frame. It has three-blade propellers measuring 6 inches. This drone sends and receives data via WiFi. Among the data that the drone sends, we can find the video stream from its monocular frontal camera and the altitude gained by fusing data from its ultrasonic sensor and barometer. Also, the camera angle can be controlled via the Software Development Kit (SDK) (https://developer.parrot.com/docs/SDK3/). Lastly, we obtain the ground truth of the position with a Vicon Vantage motion capture system (https://www.vicon.com/hardware/cameras/vantage/). We placed reflective markers on the drone and configured the capture system to deliver measurements at 100 Hz. This system provides measurements with millimeter accuracy.

## 4. Results and Discussion

This section presents results comparing the two control sub-laws described in the previous section. We begin by describing the general behavior of sensor faults induced by the ground effect. Then, the proposed strategies will be evaluated in simulation. The strategies will also be evaluated in real-world experiments. For supplementary video see: https://youtu.be/uszilXBFKP4. The project’s code is available at: https://github.com/AMatusV/mrotor-sfaults-control.

### 4.1. Altitude Sensor Faults

For low-level flights, the altitude of UAVs is measured with a combination of barometers and range sensors. The first type of sensor is affected by the ground effect because of the increment in the air pressure around the vehicle [3]. In general, the second type of sensor has limitations when there are abrupt changes in the measurement surface. Particularly, ultrasonic sensors, which are perhaps the most widely used for multirotors, are subjected to other problems such as acoustic noise and air turbulence (https://www.maxbotix.com/ultrasonic-sensor-operation-uav.htm). For the latter, the best results are obtained by mounting the sensor as far away from the propellers as possible. Nevertheless, the presence of the ground induces an upwash encountering the central part of the rotorcraft body [17,43]. Thus, flying close to the ground may cause sensor faults.

Technical manuals have reported the tendency of rotorcrafts to climb back into the air when close to the ground (https://docs.px4.io/v1.9.0/en/advanced_config/tuning_the_ecl_ekf.html, http://ardupilot.org/copter/docs/ground-effect-compensation.html). This is caused when the high-pressure zone below the drone affects the barometer. The result is a lower reading or sensor fault in pressure altitude, leading to the inner loop commanding an unwanted climb.

Applying ultrasonic wave propagation as an airflow velocity sensor is not a new concept. Ultrasonic sensors are used in several applications such as in gas, hydraulic and airflow meters [44,45]. This reinforces the idea that the ground effect could cause ultrasonic range sensor faults. Moreover, the authors’ experience has shown the following strange behavior when flying quadrotors close to the ground—the rotorcraft suddenly descends without being commanded to do so. This behavior is not in line with the literature about the ground effect.

**Figure 5:**
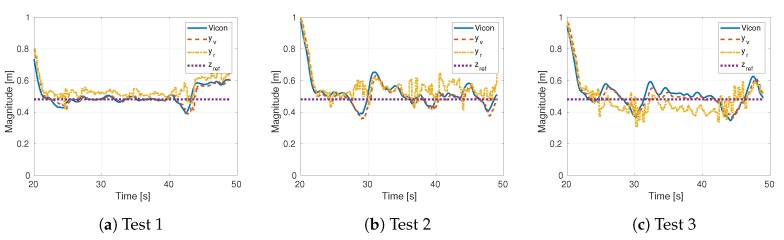
Position response while hovering close to the ground.

**Figure 6:**
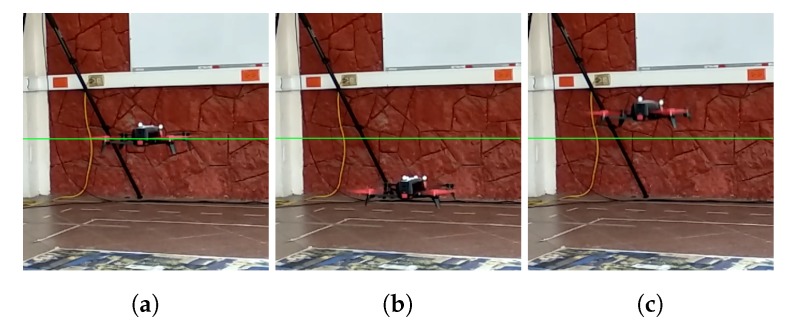
Photographs showing the quadrotor in three conditions: (**a**) faultless, (**b**) descending due to an upward sensor fault, and (**c**) ascending due to a downward sensor fault; we indicate the reference with a green line; for supplementary video check https://youtu.be/uszilXBFKP4.

To demonstrate the general behavior of the altitude faults induced by the ground effect, we collected data from the quadcopter described in Section 3.5. With a proportional outer loop controller, we commanded the drone to hover close to the ground. The feedback for this controller is obtained from the vision algorithm described in Section 3.3. Figure 5 presents the results of three tests in which sensor faults actually occur. A sensor fault occurs when the altitude reported by the drone, $y_{r}$, deviates from the ground truth (Vicon). For example, a sensor fault appeared in Test 1 around the 40 s mark which last 3 s approximately. In Test 2, a sensor fault appeared between 27 s and 30 s. Remaining deviations in this test did not affect the vehicle significantly and could be ignored by a suitable detection threshold. Test 3 shows a fault between 25 s and 30 s, the deviation was in the opposite direction than the faults in the other tests. Another fault happened around 42 s which can be detected by an appropriate weighting of the residual. Deviations detected as sensor faults cause the inner loop to react, producing a disturbance for the outer controller, see Figure 6. As expected, the position disturbance is in the opposite direction of the sensor fault.

It is important to note that the vision-based measurement agrees well with the ground truth, even though it relies on the range sensor to estimate the metric pose. The vision algorithm, assuming planar ground and knowing the camera angle and distance to the ground, constructs a synthetic depth image, which is coupled to an RGB (Red-Green-Blue) frame. Then, the RGB-D (Red-Green-Blue-Depth) version of ORB-SLAM2 consumes this data to generate a pose estimate. Our results show that the SLAM system is robust against range sensor faults when the camera moves mainly along the principal axis. In this situation, the ability to perform relocalization and reuse the map yield robustness to the system. For this to happen, the initial synthetic depth image should be generated with faultless altitude readings, which can be easily obtained while hovering far from horizontal and vertical surfaces.

Similar to this subsection, in the next ones, we will restrict to examining tests in hover conditions. This restriction is reasonable since it represents the situation in which the vehicle is most vulnerable to disturbances. Moreover, this situation extends to missions where the drone is tracking a trajectory in the *x*-*y* plane while maintaining a constant altitude.

### 4.2. Simulation

In the simulation, besides the double integrator model (4), we considered the following expression for the rotor thrust increment due to the ground effect:

(32) $$\frac{T_{IGE}}{T_{OGE}}=\frac{1}{1-\frac{R^{2}}{16z^{2}}},$$

where $T_{OGE}$ is the thrust generated by the rotorcraft flying out of ground effect, $T_{IGE}$ is the thrust when the rotorcraft is in ground effect, *R* is the radius of the rotor, and *z* is the vertical distance of the rotor to the ground. Specifically, we set R=4r, where r=0.0762 m is the radius of one propeller of the Bebop 2.

The control objective is to track a reference of 0.5 m from initial conditions of 1 m and 0 m/s. The mass of the vehicle is 0.5 kg and the gravity acceleration is 9.81 m/s^2^. The values of fault-free control parameters are $k_{p,1}=1$, $k_{d,1}=0.1$, and $k_{p,2}=k_{i,2}=10$. The residual weighting matrix is W=diag(2,0.5) and the fault detection threshold is $J_{th}=0.2$. For the FTC strategies, the parameters are $k_{a}^{+}=0.3$, $k_{a}^{-}=0.1$, $k_{e}=1.7$ and $k_{s}=0.01$. The total simulation time is 30 s. To simulate sensor faults, we considered a uniform random generator with range [0.01,0.2], which is added to the internal loop measurement at times 5 s to 6 s, 15 s to 17, and 25 s to 28 s. We set the sampling times of the external and internal loops to 0.05 s and 0.01 s, respectively. Also, we assume that the internal loop shares its measurement with the external loop every 0.2 s.

**Figure 7:**
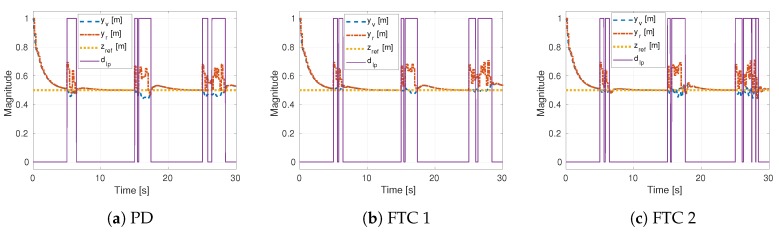
Simulation response of three control strategies.

Figure 7 shows the outputs of the sensors, the reference and the fault detection scheme. It can be seen that the sensor faults were properly detected at the time intervals at which the output of the random generator was added to the internal loop measurement. These graphical results demonstrate that the fault detector indirectly depends on the control strategy. Nevertheless, all significant sensor faults were recognized for each control strategy. Also, it can be noted that the fault can be tolerated after the control sub-laws are adopted.

**Figure 8:**
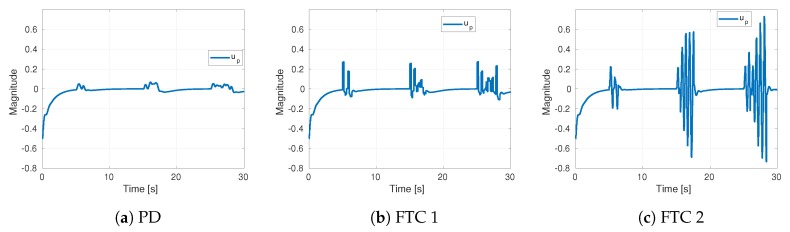
Control actions of three strategies in simulation.

Figure 8 illustrates the control actions of each strategy. As anticipated, the PD output reached lower magnitudes than the FTC strategies. On the other hand, the FTC 2 scheme attained higher command magnitudes. This might indicate problems in real implementation. The rapid changes in commands could cause fast battery depletion. Also, the vision algorithm that estimates the position could fail due to the fast movements of the camera.

Qualitatively, the FTC 2 scheme yielded the best response. For a quantitative comparison, we computed the Root Mean Square Error (RMSE) and summarized the results in Table 1. Compared to the PD controller, the proposed strategies improved the error measure in 24.41% and 38.06%, respectively.

An important take away from the simulation is the advantage of adopting asymmetrical values for $k_{a}^{+}$ and $k_{a}^{-}$. The simulation clarified that the integral action of the inner loop provides a negative offset to compensate for the ground effect. In consequence, negative commands of the external loop have more impact than positive commands until the integral reaches to zero.

### 4.3. Experiments

In the experiments, we exploit the features of the Bebop 2 to run the metric monocular SLAM algorithm to estimate the pose of the camera (refer to Section 3.3), which will be the feedback of the external controller. For all tests, the camera angle was set to −83° with respect to the horizon.

In the tests with the drone, we restrict the *x*-*y* position and the yaw angle with PID controllers: $u_{x}\left(t\right)=\mathrm{PID}\left(x;t\right)$, $u_{y}\left(t\right)=\mathrm{PID}\left(y;t\right)$, and $u_{\psi }\left(t\right)=\mathrm{PID}\left(\psi ;t\right)$. To preserve the direction in global coordinates of the *x*-*y* projection of the vector generated by the position controllers, we apply the transformation in (33).

(33) $$\begin{array}{cc} F & =\sqrt[{}]{u_{x}^{2}+u_{y}^{2}}\\ u_{x,b} & =F\cos\left[\arctan\left(\frac{u_{y}}{u_{x}}\right)-\psi \right]\\ u_{y,b} & =F\sin\left[\arctan\left(\frac{u_{y}}{u_{x}}\right)-\psi \right] \end{array}.$$

The control objective is to track a reference of 0.48 m from an initial condition of 1 m. The values of fault-free control parameters are $k_{p,1}=0.6$, and $k_{d,1}=0.2$; the drone’s inner loop parameters are kept as default. The residual weighting matrix is W=diag(1,0.5) and the fault detection threshold is $J_{th}=0.2$. For the FTC strategies, the parameters are $k_{a}^{+}=0.5$, $k_{a}^{-}=0.2$, $k_{e}=1.25$, and $k_{s}=0.01$. In practice, we observed variable frequency in the vision feedback (external loop) with an average of 15 Hz (≈ 0.0667 s). Like in the simulation, the Bebop 2 publishes altitude measurements every 0.2 s.

**Figure 9:**
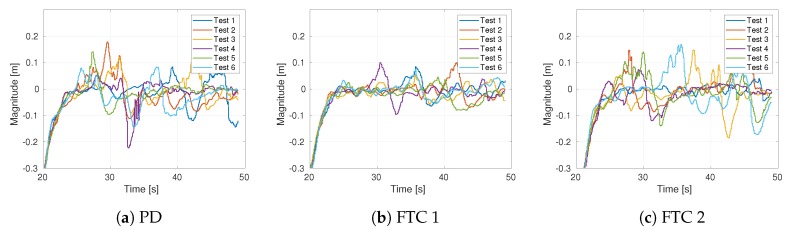
Error response of the control strategies for five tests with the real drone.

Instead of emulating the sensor faults, we opted for evaluating flights with real faults, in essence, sensor faults actually occur. Given the apparent stochastic behavior of sensor faults induced by the ground effect, we collected results from five tests for each control strategy. Only tests with detected sensor faults and without vision tracking losses were examined. For comparison, we have plotted the error signals in Figure 9. Overall, the figure reveals that the best qualitative behavior was obtained with FTC 1. This observation is confirmed with Table 2, where the FTC 1 strategy has the lowest average RMSE. Taking as reference the PD strategy, the FTC techniques improved the average error measure in 85.55% and 8.43%, respectively.

**Figure 10:**
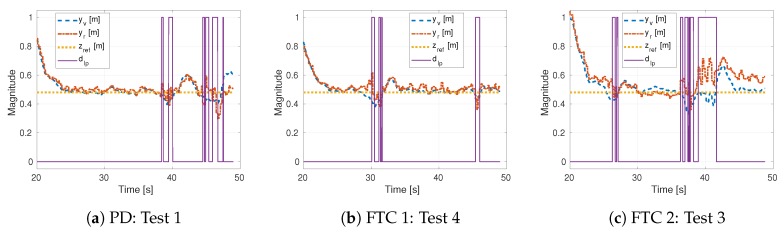
Sensors, reference and fault detection signals for individual tests.

For further comparison, we picked one test of each strategy and plotted their signals in Figure 10. Different from the simulations, in the experiments, a sensor fault may occur gradually (see the first detection in Figure 10b). In this case, a fault can still be detected (after a delay) since our detector depends on the position component of the residual. With $J_{th}$, we sacrifice the speed at which we can identify a gradual fault for robustness against noise. On the other hand, it can be observed that the worst-case scenario from FTC 1 dominates the median response of the other strategies. Unlike in the simulations, FTC 2 shows error peaks similar to the standard PD strategy.

**Figure 11:**
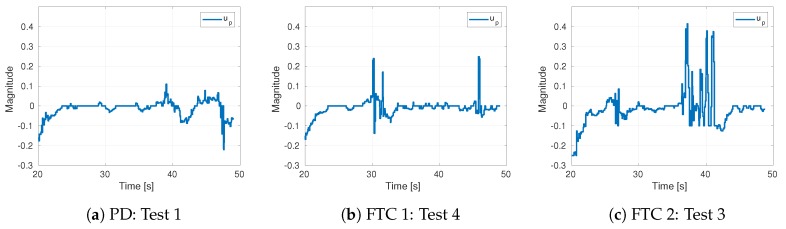
Control signals for individual tests.

The control commands for the tests in the previous plots are shown in Figure 11. The same trend as in simulations can be seen in this figure—the commands of FTC 2 exhibits the highest peaks, followed by FTC 1. The noise in the measurement and the computation of the unsmoothed first derivative explain the peaks in the PD commands.

While collecting results for each strategy, we had complications with the vision algorithm. On one hand, the vision tracking was being lost when the camera approached the ground. On the other, the vision tracking was being lost when the camera moved too fast. For the PD controller, we were losing the vision feedback because the commands allowed the drone to move close to the ground. Whereas for FTC 2, we were losing the feedback because the commands moved the drone rapidly. FTC 1 reduced the occurrence of this problem by keeping the drone close to the reference while using moderate energy.

The performance of FTC 2 was significantly different in the experiments and the simulation. One evident reason is the reduction of parameter $k_{e}$ in the experiments. We lowered this parameter because we were not obtaining tests without vision feedback losses with higher values of $k_{e}$. Another evident reason is the lower variable frequency of the real external loop. Together, these differences explain the performance degradation of FTC 2 in the experiments.

Focusing on the improvements of the error measure for FTC 1, the improvement was greater in the experiments than in the simulation. Different from the simulation, sensor faults in real tests worsen as the vehicle moves closer to the ground. This indicates that the PD controller is expected to have inferior performance in reality. Moreover, the opportune and moderate commands of FTC 1 somewhat preserved the performance of this strategy. The combined effect is the increase in error measure improvement for FTC 1 in the experiments.

## 5. Conclusions

In this article, we have presented a procedure for fault diagnosis and two control sub-laws for a multirotor with altitude sensor faults. In particular, we have considered sensor faults induced by the ground effect. In the fault-free case, a hierarchical control composed by an external PD and an internal PI controller has been developed for trajectory tracking. Exploiting the trend of onboard cameras, we have considered a vision-based feedback for the external loop based on a well known technique in robotics known as Monocular SLAM. This is the first time such a technique has been used to address any issue related to ground effect in multirotors.

The fault diagnosis has been achieved using a weighted residual, which is obtained by comparing estimations from the metric monocular SLAM system against faulty internal readings. The first control sub-law has been proposed as a combination of the external PD controller and a function of the residual. The second control sub-law has been based on SMC. The performance of the strategies has been illustrated in simulations and experiments. It is important to remark that we have adopted onboard sensors only.

Regarding the results, we have discovered that both control sub-laws overcame the performance of the standard PD controller in simulations. However, the first control sub-law offered better behavior in experiments. The performance of the second control sub-law degraded due to a combination of its rapid switching nature and limitations of the vision algorithm. In the experiments, we have found that the first control sub-law improved the RMSE in 85% compared to the PD controller.

Results reported in this work indicate that our fault detection scheme is feasible for altitude faults induced by the ground effect. To our knowledge, this is the first time that the problem of detecting internal sensor faults is addressed by using a metric monocular SLAM system. Besides, we have shown that our first control sub-law enhances the flying performance when hovering close to the ground. Therefore, this controller can be used to improve missions such as take-off, landing, hovering and operating near the ground in general.

Future work will focus on considering model uncertainties and sensor noise. These terms could be dealt with either with a state observer or with a Kalman filter. To compensate for delays in the measurements, a mathematical model could be used for prediction. Also, rejection of altitude sensor faults caused by abrupt changes in the surface below the drone should be explored.

## Figures and Tables

**Table 1:** Error measures of the control strategies in simulation.

	PD	FTC 1	FTC 2
RMSE	0.0214	0.0172	0.0155

**Table 2:** Error measures of the control strategies with the real drone.

RMSE	Test 1	Test 2	Test 3	Test 4	Test 5	Average
**PD**	0.0492	0.0591	0.0479	0.0460	0.0549	0.0514
**FTC 1**	0.0266	0.0304	0.0249	0.0317	0.0247	0.0277
**FTC 2**	0.0215	0.0576	0.0579	0.0417	0.0582	0.0474

## List of Symbols

*x*, *y*, *z*	Position in the world-fixed frame
ϕ, θ, ψ	Roll, pitch, and yaw angles
$v_{z}$	Velocity of the *z* axis
*m*	Mass of the multirotor
*g*	Gravitational acceleration
$J_{x}$, $J_{y}$, $J_{z}$	Diagonal elements of the inertia matrix
$u_{z}$	Thrust force
$u_{\phi }$, $u_{\theta }$, $u_{\psi }$	Input torques
$u_{v}$, $u_{p}$	Control inputs of acceleration and velocity
$z_{ref}$, $v_{ref}$	Position and velocity references of the *z* axis
$e_{z}$, $e_{v}$	Position and velocity errors of the *z* axis
$k_{p,1}$, $k_{d,1}$	Parameters of the external controller
$k_{p,2}$, $k_{i,2}$, $k_{d,2}$	Parameter of the internal controller
*h*	Height above ground of the camera
α	Angle of the camera
*f*	Focal length of the camera
*l*	Vector from the camera’s optical center through a pixel to the ground
*n*	Vector perpendicular to the ground from the camera’s optical center to the ground
*d*	Distance at which *l* intersects the ground
δ	Sensor fault
$y_{r}$, $y_{v}$	Range-based and vision-based measurements of the altitude
$y_{i}$	Inertial-based measurement
*r*	Residual vector
$J_{eval}$	Residual evaluation function
$J_{th}$, $d_{lp}$	Fault detection threshold and logical variable
$T_{p}$, $T_{v}$	Sampling periods of the external and internal loops
$u_{p}^{n}$, $u_{p}^{f}$	Control input of velocity in normal case and fault case
$u_{a}$	Additive control term
$k_{a}=\left\{k_{a}^{+},k_{a}^{-}\right\}$	Control gain (for positive and negative disturbances) of FTC 1
*c*, *C*	Composite disturbance term and its bound
*s*, $k_{e}$	Sliding surface and its parameter
$k_{s}$	Sliding mode control gain of FTC 2

## References

[1] Hesch J.A., Kottas D.G., Bowman S.L., Roumeliotis S.I. (2013). Camera-IMU-based localization: Observability analysis and consistency improvement. Int. J. Rob. Res..

[2] Moon H., Martinez-Carranza J., Cieslewski T., Faessler M., Falanga D., Simovic A., Scaramuzza D., Li S., Ozo M., Wagter C.D. (2019). Challenges and implemented technologies used in autonomous drone racing. Intell. Serv. Rob..

[3] Nakanishi H., Kanata S., Sawaragi T. Measurement model of barometer in ground effect of unmanned helicopter and its application to estimate terrain clearance. Proceedings of the 2011 IEEE International Symposium on Safety, Security, and Rescue Robotics.

[4] Cheeseman I., Bennet W. (1957). The Effect of the Ground on a Helicopter Rotor in Forward Flight. Aeronaut. Rese. Counc. Rep. Memo.

[5] Hayden J.S. The Effect of the Ground on Helicopter Hovering Power Required. Proceedings of the 32nd Annual Forum of the American Helicopter Society.

[6] Lee T.E., Leishman J.G., Ramasamy M. (2010). Fluid Dynamics of Interacting Blade Tip Vortices with a Ground Plane. J. Am. Helicopter Soc..

[7] Nonaka K., Sugizaki H. Integral sliding mode altitude control for a small model helicopter with ground effect compensation. Proceedings of the 2011 American Control Conference.

[8] Ryan T., Kim H.J. Modelling of Quadrotor Ground Effect Forces via Simple Visual Feedback and Support Vector Regression. Proceedings of the AIAA Guidance, Navigation, and Control Conference.

[9] Lee D., Kim H.J., Sastry S. (2009). Feedback linearization vs. adaptive sliding mode control for a quadrotor helicopter. Int. J. Control Autom. Syst..

[10] Mirzaei M., Nia F.S., Mohammadi H. Applying adaptive fuzzy sliding mode control to an underactuated system. Proceedings of the 2nd International Conference on Control, Instrumentation and Automation.

[11] Matus-Vargas A., Rodriguez-Gomez G., Martinez-Carranza J. Aerodynamic Disturbance Rejection Acting on a Quadcopter Near Ground. Proceedings of the 2019 6th International Conference on Control, Decision and Information Technologies (CoDIT).

[12] Robinson D.C. (2016). Modelling and Estimation of Aerodynamic Disturbances Acting on a Hovering Micro Helicopter in Close Proximity to Planar Surfaces. Ph.D. Thesis.

[13] Lakshminarayan V.K., Kalra T.S., Baeder J.D. (2013). Detailed Computational Investigation of a Hovering Microscale Rotor in Ground Effect. AIAA J..

[14] Kalra T.S. (2014). CFD Modeling and Analysis of Rotor Wake in Hover Interacting with a Ground Plane. Ph.D. Thesis.

[15] Guenard N., Hamel T., Eck L. Control Laws For The Tele Operation of An Unmanned Aerial Vehicle Known as An X4-flyer. Proceedings of the 2006 IEEE/RSJ International Conference on Intelligent Robots and Systems.

[16] Powers C., Mellinger D., Kushleyev A., Kothmann B., Kumar V. (2013). Influence of Aerodynamics and Proximity Effects in Quadrotor Flight. Experimental Robotics.

[17] Sharf I., Nahon M., Harmat A., Khan W., Michini M., Speal N., Trentini M., Tsadok T., Wang T. Ground effect experiments and model validation with Draganflyer X8 rotorcraft. Proceedings of the 2014 International Conference on Unmanned Aircraft Systems (ICUAS).

[18] Sanchez-Cuevas P., Heredia G., Ollero A. (2017). Characterization of the Aerodynamic Ground Effect and Its Influence in Multirotor Control. Int. J. Aerosp. Eng..

[19] Nobahari H., Sharifi A. (2014). Continuous ant colony filter applied to online estimation and compensation of ground effect in automatic landing of quadrotor. Eng. Appl. Artif. Intell..

[20] Hu B., Lu L., Mishra S. Fast, safe and precise landing of a quadrotor on an oscillating platform. Proceedings of the 2015 American Control Conference (ACC).

[21] Danjun L., Yan Z., Zongying S., Geng L. Autonomous landing of quadrotor based on ground effect modelling. Proceedings of the 2015 34th Chinese Control Conference (CCC).

[22] Bartholomew J., Calway A., Mayol-Cuevas W. Learning to predict obstacle aerodynamics from depth images for Micro Air Vehicles. Proceedings of the 2014 IEEE International Conference on Robotics and Automation (ICRA).

[23] Bartholomew J., Calway A., Mayol-Cuevas W. Improving MAV control by predicting aerodynamic effects of obstacles. Proceedings of the 2015 IEEE/RSJ International Conference on Intelligent Robots and Systems (IROS).

[24] Tomic T., Haddadin S. A unified framework for external wrench estimation, interaction control and collision reflexes for flying robots. Proceedings of the 2014 IEEE/RSJ International Conference on Intelligent Robots and Systems.

[25] Du H., Pu Z., Yi J., Qian H. Advanced quadrotor takeoff control based on incremental nonlinear dynamic inversion and integral extended state observer. Proceedings of the 2016 IEEE Chinese Guidance, Navigation and Control Conference (CGNCC).

[26] Robinson D.C., Ryan K., Chung H. Helicopter hovering attitude control using a direct feedthrough simultaneous state and disturbance observer. Proceedings of the 2015 IEEE Conference on Control Applications (CCA).

[27] Xiao B., Yin S. (2017). A New Disturbance Attenuation Control Scheme for Quadrotor Unmanned Aerial Vehicles. IEEE Trans. Ind. Inf..

[28] McKinnon C.D., Schoellig A.P. Unscented external force and torque estimation for quadrotors. Proceedings of the 2016 IEEE/RSJ International Conference on Intelligent Robots and Systems (IROS).

[29] Zhang Y., Chamseddine A., Rabbath C., Gordon B., Su C.Y., Rakheja S., Fulford C., Apkarian J., Gosselin P. (2013). Development of advanced FDD and FTC techniques with application to an unmanned quadrotor helicopter testbed. J. Franklin Inst..

[30] Berbra C., Lesecq S., Martinez J. A Multi-observer Switching Strategy for Fault-Tolerant Control of a Quadrotor Helicopter. Proceedings of the 2016 16th Mediterranean Conference on Control and Automation.

[31] Rafaralahy H., Richard E., Boutayeb M., Zasadzinski M. Simultaneous observer based sensor diagnosis and speed estimation of Unmanned Aerial Vehicle. Proceedings of the 2008 47th IEEE Conference on Decision and Control.

[32] Nguyen H., Berbra C., Lesecq S., Gentil S., Barraud A., Godin C. Diagnosis of an Inertial Measurement Unit based on set membership estimation. Proceedings of the 2009 17th Mediterranean Conference on Control and Automation.

[33] Qin L., He X., Yan R., Zhou D. (2017). Active Fault-Tolerant Control for a Quadrotor with Sensor Faults. J. Intell. Rob. Syst..

[34] Cook M.V. (2013). Systems of Axes and Notation. Flight Dynamics Principles.

[35] Bouabdallah S., Siegwart R. Full control of a quadrotor. Proceedings of the 2007 IEEE/RSJ International Conference on Intelligent Robots and Systems.

[36] Bresciani T. (2008). Modelling, Identification and Control of a Quadrotor Helicopter. Master’s Thesis.

[37] Xuan-Mung N., Hong S.K. (2019). Improved Altitude Control Algorithm for Quadcopter Unmanned Aerial Vehicles. Appl. Sci..

[38] Khan H.S., Kadri M.B. Attitude and altitude control of quadrotor by discrete PID control and non-linear model predictive control. Proceedings of the 2015 International Conference on Information and Communication Technologies (ICICT).

[39] Mur-Artal R., Tardos J.D. (2017). ORB-SLAM2: An Open-Source SLAM System for Monocular, Stereo, and RGB-D Cameras. IEEE Trans. Rob..

[40] Noura H., Theilliol D., Ponsart J.C., Chamseddine A. (2009). Fault-tolerant Control Systems.

[41] Tan C.P., Edwards C. (2003). Sliding mode observers for robust detection and reconstruction of actuator and sensor faults. Int. J. Robust Nonlinear Control.

[42] Zhang K., Jiang B., Cocquempot V. (2008). Adaptive Observer-based Fast Fault Estimation. Int. J. Control Autom. Syst..

[43] Griffiths D.A., Ananthan S., Leishman J.G. (2005). Predictions of Rotor Performance in Ground Effect Using a Free-Vortex Wake Model. J. Am. Helicopter Soc..

[44] Raine A., Aslam N., Underwood C., Danaher S. (2015). Development of an Ultrasonic Airflow Measurement Device for Ducted Air. Sensors.

[45] Davies D.G., Bolam R.C., Vagapov Y., Excell P. Ultrasonic sensor for UAV flight navigation. Proceedings of the 2018 25th International Workshop on Electric Drives: Optimization in Control of Electric Drives (IWED).

